# Genetic association of *LPL* rs1121923 and rs258 with plasma TG and VLDL levels

**DOI:** 10.1038/s41598-019-42021-3

**Published:** 2019-04-03

**Authors:** Suzanne A. Al-Bustan, Ahmad Al-Serri, Majed A. Alnaqeeb, Babitha G. Annice, Olusegun Mojiminiyi

**Affiliations:** 1 Department of Biological Sciences, Faculty of Science, Kuwait University, Kuwait, Kuwait; 2 Unit of Human Genetics, Department of Pathology, Faculty of Medicine, Kuwait University, Kuwait, Kuwait; 3 Mubark Al-Kabeer Hospital, Ministry of Health, Kuwait, Kuwait

## Abstract

Lipoprotein lipase (*LPL*) is a rate-limiting enzyme for the hydrolysis of triglycerides (TG). Hundreds of genetic variants including single nucleotide polymorphisms have been identified across the 30Kb gene locus on chromosome 8q22. Several of these variants have been demonstrated to have genetic association with lipid level variation but many remain unresolved. Controversial reports on the genetic association of variants among different populations pose a challenge to which variants are informative. This study aimed to investigate “common” *LPL* variants (rs1121923, rs258, rs328, rs13702) and their possible role in plasma lipid level. Genotyping was performed using Realtime PCR. Based on the observed genotypes, the minor allele frequencies were *A*: 0.065 for rs1121923; *C*: 0.379 for rs258; *G*: 0.087 for rs328 and *C*: 0.337 for rs13702. Using linear regression, a lowering effect of rs1121923 (p = 0.024) on TG levels (−0.14 *B* coefficient: CI: −0.27–−0.019) and rs258 (p = 0.013) on VLDL levels (*B*: −0.046; CI: −0.082–−0.009) was observed indicating a “protective” role for the two variants. Moreover, the findings indicate the potential for including rs1121923 and rs258 in diagnostic panels for use as an estimator of “risk” scores for dyslipidemia.

## Introduction

Plasma lipid levels play an important role in maintaining homeostasis and are useful risk markers for cardio-metabolic disorders such as the metabolic syndrome and type 2 diabetes mellitus (T2DM). Persistent elevation of plasma lipid levels often results in dyslipidemia that may lead to further complications such as coronary heart disease (CHD). It has been reported that hypertriglyceridemia (HTG) is a risk factor for CHD through mediating decreased levels of high density lipoprotein-cholesterol (HDL-C) and increased levels of low density lipoprotein-C (LDL-C) that may facilitate thrombogenicity leading to atherosclerosis^1^. Although numerous studies have demonstrated the complex etiology of dyslipidemia implicating a variety of environmental factors such as nutrition, conflicting results persist with regards to the role of genetic factors. More recently, studies have indicated that the heritably of plasma lipid levels is not only influenced by numerous genetic variants but also by ethnicity^2–6^. In fact, Deo *et al*. reported that local ancestry contributed significantly (p < 0.05) to variation in lipid levels^3^. Furthermore, Johansen *et al*. (2010) indicated that both common and rare genetic variants could explain 41.6% of total variation in HTG with common genetic variants explaining 20.8% (specifically for 7 gene loci) and the rare genetic variants explaining only 1.1% (at 4 gene loci) while the other associated factors explained 19.7% of the cases^7^. Among all these studies, the gene for lipoprotein lipase (*LPL*) has always been implicated with a significant influence on one or multiple variations in lipid parameters^2–4,6–9^.

The *LPL* gene codes for the 475-amino acid enzyme responsible for the hydrolysis of triglycerides to free fatty acid and is an important catalyst in lipid metabolism and transport pathways. The gene has been fully mapped to chromosome 8q22 and has been fully sequenced in different ethnic groups^2,5,6,10–12^. The gene spans 30Kb including 10 exons with the first exon encoding the 5′ untranslated region (UTR) and signal peptide and the last exon encoding the full 3′ UTR. Most of the gene comprises noncoding sequences mainly localized to intron 1^6,10^. Some studies have reported associations of numerous variants in both the coding and the non-coding regions, especially in introns 2, 3, 5, 6 and 8 with variation in lipid levels^2,5,6,10–15^.

Despite consistency in the implication of *LPL* and variation in lipid levels with associated clinical manifestations^2,16–22^, the effect of many of the significant variants identified were not observed in different populations and ethnic groups^2,3,5,6,8,9,12,13^. It has been suggested that the role of *LPL* and its variants be further investigated in different populations with reference to ethnic backgrounds^2^. However, challenges are encountered when deciding which variants should be selected for genetic association with lipid levels^3,6,7,9^. A common approach would be to select a representative SNP that is in strong Linkage disequilibrium with other SNPs and that the selected tagged SNPs would represent the different haplogroups across the *LPL* gene. Such an approach may prove to be time and cost ineffective. Johansen *et al*. (2010) indicated that 20.8% of variation in TG levels could be explained by common variants. In addition, Deo *et al*. identified 12 risk variants at the *LPL* gene locus associated with TG levels, four of which are included in this study. Moreover, Evans *et al*. (2013) suggested the use of the common disease common variant (CDCV) model is applicable to studying *LPL* variants. The model implies that genetic variants which occur at high frequencies among the general population would increase the susceptibility to the disease but with a small “effect size”^7^.

With this approach in mind, the present study aimed to investigate common *LPL* variants with a global minor frequency above 5% among the general Kuwaiti population to assess their role in contributing to fluctuations in plasma lipid levels, specifically triglycerides (TG) and high-density lipoprotein-Cholesterol (HDL-C). The four variants selected have been previously reported for their effect size in different ethnic backgrounds^3,5,12^. Two of the variants selected (rs1121923 and rs258) have not been extensively studied in different populations^21,22^ while the other two (rs328 and rs13702) have been extensively investigated with conflicting results^2–5,8,22^. Deo *et al*. (2009) identified rs328 as a strong indicator (p = 2.7 × 10^−6^) of increased TG levels with a higher impact in populations of African ancestry when compared to Europeans. The other commonly studied variant rs325 was not included as it showed strong LD with rs328. However, conflicting results have been reported with regards to rs328^3–5,12,22^. Several studies have suggested that rs13702 is a strong candidate for TG and HDL-C and TG levels^4,8,23^.

The present study investigated *LPL* common variants (rs1121923; rs258; rs328 and rs13702) (Table 1) in a Kuwaiti cohort that have been implicated in disorders associated with plasma lipid levels and that yielded either conflicting or inconclusive findings. The SNPs are in regions reported to be involved in gene expression or splice regulation. Common variants, based on the minor allele frequency reported in the GenBank database and based on Genome Reference Consortium Human Build 38 patch release 12 (Grch38.p12), are more likely to indicate a genetic association with variation in lipid levels, if present, among a small sample size which is representative of a heterogenous population. The cohort in this study is a representative of an Arab admixed population^24^ in which common variants are more likely to yield informative and conclusive results than rare variants among a population known for demonstrating high prevalence of dyslipidemia and the metabolic syndrome^25–27^.

**Table 1:** *LPL* variants selected for this study, their position and characteristics.

SNP	Alleles	Gene Location	Gene Position	Genomic Location	Consequence	Global MAF
rs1121923	G > A	Exon 3	17854	8:19951924–19951924	synonymous_ variant Modifier impact	A: 0.05
rs258	G > C	Intron 5	20671	8:19954741–19954741	intron_variant Modifier impact	C: 0.44
rs328	C > G	Exon 9	28143	8:19962213–19962213	stop gained/regulatory region variant^15^ High impact	G: 0.09
rs13702	T > C	3′UTR	32911	8:19966981–19966981	3_prime_UTR_variant Gain of Function^19^ Modifier Impact	C: 0.33

The information was obtained from Genome Reference Consortium Human Build 38 patch release 12 (Grch38.p12).

## Results

### Genotyping, Hardy-Weinberg Equilibrium and Linkage Disequilibrium

The most frequent genotype observed was that for the homozygous wildtype allele except for variant rs258 in which the heterozygous form had the highest frequencies (Table 2). All the frequencies were found to be in HWE (p > 0.05). The MAF was found to be lowest for rs1121923 *A* allele (A: 0.065) while the highest was for rs258 (C: 0.379).

**Table 2:** Genotype and allele frequencies of the four studied *LPL* variants among the cohort (n = 702).

*LPL* Variant	Genotype Frequency			MAF	HWE P value
	WW	WM	MM		
rs1121923G < A exon 3	0.875 (n = 623)	0.121 (n = 86)	0.004 (n = 3)	A: 0.065	0.9861
rs258G < C intron 5	0.355 (n = 282)	0.465 (n = 321)	0.180 (n = 109)	C: 0.379	0.2653
rs328C < G exon 9	0.830 (n = 591)	0.166 (n = 118)	0.004 (n = 3)	G: 0.087	0.2581
rs13702T < C 3′UTR	0.446 (n = 318)	0.433 (n = 308)	0.121 (n = 86)	C: 0.337	0.3922

^*^W = wildtype (ancestral) allele, M = minor allele MAF: Minor Allele Frequency

HWE: Hardy-Weinberg Equilibrium.

**Figure 1:**
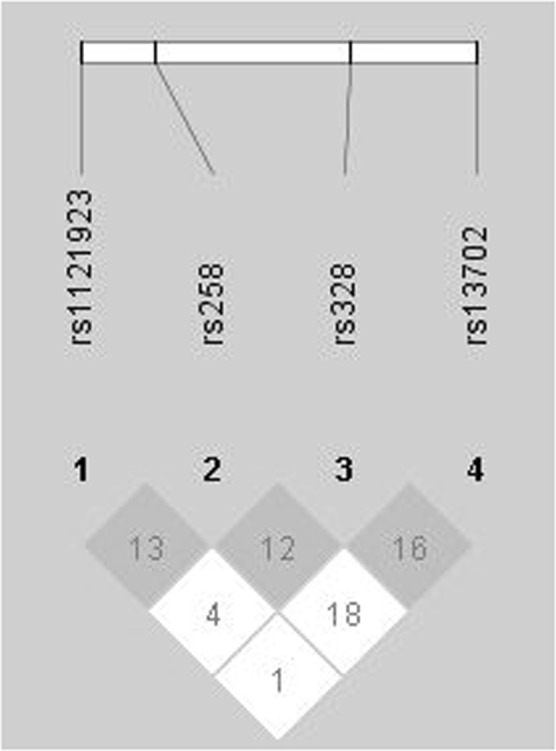
Haploblock scheme between *LPL* rs1121923, rs258, rs328 and rs13702 based on linkage disequilibrium and recombination frequencies. The confidence Bounds Color Scheme shows evidence of LD (dark grey, black), uninformative (shades of light grey) and strong evidence of recombination (white) based on the r^2^ values, all of which were found insignificant. The image was generated by Haploview (version 4.2.).

Analysis of LD revealed that the four selected variants were not correlated and didn’t form a common haploblock (Fig. 1). The four SNPs at the *LPL* locus (rs1121923, rs258, rs328 and rs13702) were analyzed in a total of 12 pair-wise combinations and the resulting r^2^ values showed no significant LD between any SNP pairs (r^2^ < 0.8).

### Genetic Association and Regression Analysis

Linear regression analysis based on the additive genetic model of the four variants against variation in plasma lipid levels detected significant associations (p < 0.05) for only rs1121923 (Table 3) and rs258 with TG and VLDL levels respectively (Table 4). Significant (p = 0.02), however the significance level was comprised after Bonferroni’s correction (p = 0.0125), lower levels of TG (0.94 mmol/L ± 0.74 for GA and 1.09 mmol/L ± 0.84 for GG) and border line significance of VLDL (0.38 mmol/L ± 0.30 for GA and 0.45 mmol/L ± 0.35 for G) were observed for the heterozygous individuals. In addition, significantly lower VLDL levels were observed in both heterozygous (0.41 mmol/L ± 0.31) and homozygous individuals for the minor allele *C* (0.43 mmol/L ± 0.27) when compared to the homozygous wildtype allele G (0.48 mmol/L ± 0.41) for rs258. A borderline significant association was also observed for rs328 (Table 5) with LDL-C levels in which individuals with the homozygous wildtype had significantly lower levels (3.09 mmol/L ± 0.81) than carriers (3.24 mmol/L ± 0.86 for GC and 3.2 mmol/L ± 0.52 for GG) of the minor *G* allele. No significant association was observed for rs13702 (Table 6).

**Table 3:** Association of *LPL* rs1121923 with plasma lipid levels (expressed in mmol/L) in the cohort (n = 702) using the additive genetic model.

Variable	Genotype	Number	Mean	*B*-coefficient (95% CI)	p-value
TC	GG	614	4.74 ± 0.95	1	0.89
	GA	85	4.67 ± 0.88	−0.01 (−0.24–0.22)	
	AA	3	3.66 ± 1.11	−0.18 (−1.95–1.58)	
HDL	GG	597	1.12 ± 0.32	1	0.11
	GA	82	1.18 ± 0.29	0.06 (−0.01–0.14)	
	AA	3	1.11 ± 0.17	−0.05 (−0.63–0.53)	
LDL	GG	595	3.13 ± 0.81	1	0.92
	GA	82	3.08 ± 0.84	0.007 (−0.19–0.21)	
	AA	3	2.26 ± 1.13	0.06 (−1.45–1.57)	
TG	GG	614	1.09 ± 0.84	1	**0.02**
	GA	85	0.94 ± 0.74	−0.14 (−0.27, −0.01)	
	AA	3	0.61 ± 0.14	−0.42 (−1.42–0.57)	
VLDL	GG	614	0.45 ± 0.35	1	0.05
	GA	85	0.38 ± 0.30	−0.06 (−0.14–0.006)	
	AA	3	0.24 ± 0.05	−0.2 (−0.77–0.37)

**Table 4:** Association of *LPL* rs258 with plasma lipid levels (expressed in mmol/L) in the cohort (n = 702) using the additive genetic model.

Variable	Genotype	Number	Mean	*B*-coefficient (95% CI)	p-value
TC	GG	278	4.75 ± 1.01	1	0.62
	GC	315	4.69 ± 0.91	−0.04 (−0.22,−0.12)	
	CC	109	4.75 ± 0.87	−0.11 (−0.34, 0.11)	
HDL	GG	268	1.11 ± 0.33	1	0.45
	GC	305	1.14 ± 0.3	0.02 (−0.035, 0.08)	
	CC	109	1.13 ± 0.31	−0.02 (−0.1, 0.05)	
LDL	GG	267	3.11 ± 0.81	1	0.83
	GC	304	3.12 ± 0.83	0.02 (−0.12, 0.18)	
	CC	109	3.13 ± 0.8	−0.03 (−0.23, 0.16)	
TG	GG	278	1.15 ± 0.96	1	0.17
	GC	315	1 ± 0.74	−0.09 (−0.19, 0.008)	
	CC	109	1.05 ± 0.66	−0.07 (−0.21, 0.05)	
VLDL	GG	278	0.48 ± 0.41	1	**0.01**
	GC	315	0.41 ± 0.31	−0.06 (−0.12 −0.006)	
	CC	109	0.43 ± 0.27	−0.08 (−0.15–0.008)

**Table 5:** Association of *LPL* rs328 with plasma lipid levels (expressed in mmol/L) in the cohort (n = 702) using the additive genetic model.

Variable	Genotype	Number	Mean	*B*-coefficient (95% CI)	p- value
TC	CC	582	4.7 ± 0.96	1	0.16
	CG	113	4.83 ± 0.88	0.12 (−0.08–0.33)	
	GG	3	4.9 ± 0.62	0.66 (−0.58 1.91)	
HDL	CC	566	1.12 ± 0.32	1	0.76
	CG	113	1.14 ± 1.08	0.007 (−0.06–0.07)	
	GG	3	1.08 ± 0.31	0.08 (−0.33–0.49)	
LDL	CC	564	3.09 ± 0.81	1	0.05
	CG	113	3.24 ± 0.86	0.16 (−0.02–0.34)	
	GG	3	3.2 ± 0.52	0.52 (−0.53–1.59)	
TG	CC	582	1.08 ± 0.86	1	0.34
	CG	117	0.98 ± 0.6	−0.07 (−0.19–0.04)	
	GG	3	1.29 ± 0.76	0.23 (−0.47–0.94)	
VLDL	CC	582	0.44 ± 0.36	1	0.11
	CG	117	0.4 ± 0.24	−0.06 (−0.13–0.008)	
	GG	3	0.52 ± 0.3	0.02 (−0.38–0.43)

**Table 6:** Association of *LPL* rs13702 with plasma lipid levels (expressed in mmol/L) in the cohort (n = 702) using the additive genetic model.

Variable	Genotype	Number	Mean	*B*-coefficient	p-value
				(95% CI)	
TC	TT	311	4.66 ± 0.93	1	0.7
	TC	305	4.76 ± 0.94	0.01 (−0.15–0.18)	
	CC	86	4.81 ± 1.03	0.05 (−0.2–0.31)	
HDL	TT	303	1.11 ± 0.34	1	0.71
	TC	295	1.13 ± 0.31	−0.006 (−0.06–0.05)	
	CC	84	1.13 ± 0.28	−0.01 (−0.1–0.07)	
LDL	TT	301	3.03 ± 0.76	1	0.5
	TC	295	3.19 ± 0.86	0.06 (−0.08–0.21)	
	CC	84	3.17 ± 0.83	0.03 (−0.18−0.26)	
TG	TT	311	1.13 ± 0.96	1	0.45
	TC	305	1 ± 0.64	−0.01 (−0.11–0.08)	
	CC	86	1.05 ± 0.86	−0.06 (−0.2–0.08)	
VLDL	TT	311	0.47 ± 0.4	1	0.36
	TC	305	0.41 ± 0.26	−0.03 (−0.08–0.02)	
	CC	86	0.44 ± 0.39	−0.02 (−0.1–0.06)	

A multivariate analysis using linear regression was conducted on the significant SNPs to indicate the predictor variables associated with lipid levels. BMI, age and gender along with rs1121923 genotype were all found to be predictors of TG levels (Table 7). The AA genotype of rs258 was associated with a lowering effect indicated by a *B* coefficient of −0.14 (95% CI: −0.27 – −0.019; p = 0.24). In addition, BMI, age and gender along with rs1121923 were significantly associated with VLDL levels (Table 8). The genotype CC of rs258 was associated with a lowering effect as indicated by a *B* coefficient of −0.046 (95% CI: −0.082 – −0.009; p = 0.013).

**Table 7:** Multivariate analysis to predict factors including rs1121923 associated with TG (log transformed) in the studied cohort (n = 702).

Variable	*B*-coefficient (95% CI)	p-value
BMI	1.024 (1.017–1.031)	<0.0001
Age	1.015 (1.011–1.018)	<0.0001
Sex	0.971 (0.72–0.86)	<0.0001
*LPL* rs1121923	−0.14 (−0.27–−0.019)	0.024

**Table 8:** Multivariate analysis to predict factors including rs258 associated with VLDL levels (log transformed) in the studied cohort (n = 702).

Variable	*B*-coefficient (95% CI)	p-value
BMI	1.01 (1.006–1.014)	<0.0001
Age	1 (1.004–1.008)	<0.0001
Sex	0.9 (0.85–0.95)	<0.005
*LPL* rs258	−0.046 (−0.082–−0.009)	0.013

## Discussion

The present study is the first to report a significant association of an *LPL* intronic variant rs258 with a lowering effect on VLDL levels as well as a lowering effect of the synonymous variant rs1121923 on plasma TG levels amongst an apparently healthy individual. Both variants have been previously implicated for their potential effects on LPL activity and role in affecting plasma lipid levels^3,6,22^. However, the novel findings from the present study clearly demonstrated the significant “protective” effect of the minor alleles of the two variants in lowering TG and VLDL levels.

The positive association of these non-structural variants may be the outcome of their interaction with other loci that modulate LPL expression levels. It has been suggested that although the synonymous variant rs1121923 located in exon 3 doesn’t alter the enzyme structure, it may however affect LPL levels^22,28^ and/or activity. In a recent study, a strong positive association of these variants was reported with higher HDL-C levels for carriers of the minor allele^21^. The present study also supports a “protective” effect of rs1121923 variants on plasma lipid levels despite the effect of Bonferroni’s correction on the initial significance observed (p = 0.02). It worth noting that there are concerns when applying Bonforroni’s correction especially since it relies on the assumption that the same variants are all simultaneously significant which is not the case in this type of study in addition to its contribution in increasing Type II errors^29^. The haplotype analysis clearly demonstrated that the four variants are segregating independently. In addition, the authors opted to perform haplotype analysis that takes into consideration several significant variants that may occur simultaneously but through linkage disequilibrium.

The mechanism by which the minor allele of this variant lower’s TG is more likely to be the outcome of interaction with other variants either at the same gene locus or other gene loci involved in lipid metabolism and transport. It has been suggested that simple variants within regions encompassing consensus and important sequences may exert a pathogenic effect through the inactivation of splice and/or activation of cryptic splice sites leading to undesirable alternative splicing^2^. This is a likely scenario for rs1121923 as exon three codes for a fraction of the 20 amino acids that form the B loop of the LPL enzyme, which is important for its catalytic activity^30^.

It is worth pointing out that the novel findings in the present study is in establishing a significant association of *LPL*, and specifically variant rs258, with variation in VLDL levels. Other studies have reported an association of other variants of *LPL* with VLDL^14,31^. Salinelli *et al*. (1998) demonstrated the role the LPL enzyme plays in the uptake of VLDL followed by its hydrolysis through the binding of specific domains in the enzyme to a lipoprotein receptor^14^. The association of rs258 with its lowering effect on VLDL level may also be through affecting protein binding and/or regulation of expression levels of *LPL* similar to other reported intronic variants^17,32^. A few studies have reported novel and rare variants in intronic regions to be associated with VLDL levels^6,33^. Both rs261 and rs263 in intron 5 have been reported to be associated with changes in both TG and HDL levels^3^ thus supporting the effect variants might have on plasma lipid levels. The mechanism behind this effect may be due to intron 5 harboring sites for regulatory elements as has been observed with intron 8 and the effect of rs325^15^. Another important consideration is that intron 5 flanks the coding sequences for the binding sites of APOC2 and that can affect the catalytic activity of the enzyme^11^. Intron 5 has the least number of reported variants as compared to intron 1, 6 and 9 indicating that it has highly conserved regions due to its important regulatory role and/or due to the relative size of this intron. It has been suggested that regulatory elements, believed to span across the *LPL* gene locus at the 3′ and 5′ UTR as well as within intronic regions, could be sensitive to *trans* acting regulatory factors which may be either intrinsic or extrinsic^15^. This may affect the modulation of binding of transcription factors needed during gene expression, and in turn contribute to the risk of dyslipidemia and the subsequent clinical manifestation of the metabolic syndrome and CHD^6,19,20^.

Although several studies have reported positive genetic association of rs328 and rs13702^3,5,8,17,21,22^ with plasma lipid levels, the present study did not identify any such association. This is very likely a consequence of choosing apparently healthy subjects in the studied cohort. Both rs328 and rs13702 variants have been documented to be associated with a pathogenic effect increasing the risk to clinical dyslipidemia and the metabolic syndrome. Another interesting finding is the detected association between the two *LPL* variants (rs1121923 and rs258) is likely to be the outcome of the number of heterozygous individuals since very low numbers of homozygous for the minor allele were identified in the cohort. This suggests that the alleles at these two loci are co-dominant which is supported by the additive genetic modeling (Tables 7 and 8). Accordingly, it is likely that the pathogenic potential of these two variants may be observed in clinical cases of dyslipidemia, the metabolic syndrome and CHD where there may be a higher frequency of homozygosity for the minor allele than individuals devoid of such diseases.

Studies that supported a genetic association with lipid levels included numerous *LPL* variants and tagged SNPs, based on the haplogroups, that included rs13702, rs320, rs325 and rs328. These studies had excluded rs1121923 and rs258 from the association studies which in this study were identified to be worthy of analysis along with rs13702 and rs328 (Table 9). In one study that reported a significant association of rs320 with TG levels in Hispanics^28^ was often in linkage disequilibrium with rs328^3,12^ suggested that some variants maybe independent of other variable sites in the *LPL* gene locus. This supports the haplotype analysis in this study which revealed that the four selected variants (Fig. 1) are not in linkage disequilibrium thus suggesting that rs1121923 and rs258 a high direct and an independent association with TG and VLDL levels.

**Table 9:** Reported minor allele frequencies (MAF) with the four selected SNPs among different populations.

Variant MAF/population	rs1121923 (Exon 3) A allele	rs258 (Intron 5) C allele	rs328 (Exon 9) G allele	rs13702 (3′UTR) C allele
Global*	0.0491	0.5593	0.0925	0.3349
Africans*	0.1392	0.9327	0.0613	0.5371
Americans*	0.0274	0.4294	0.0634	0.2810
East Asians*	0.0409	0.3700	0.1220	0.2321
Europeans*	0.0398	0.4732	0.1302	0.3201
South East Asians*	0.0031	0.4305	0.0859	0.2209
African Americans*	0.1139	NA	NA	NA
Chinese^2^	NA	NA	0.056 (HTG) 0.072 (Control)	NA
Europeans^3^	0.02	0.40	0.86	0.66
Africans^3^	0.13	0.96	0.94	0.43
African Americans^6^	NA	NA	NA	0.459
Germans^8^	NA	NA	NA	0.071 (HTG) 0.319 (LTG)
Americans^9^	NA	NA	0.07	NA
Non-Hispanic Whites^12^	NA	NA	0.103	0.272
United Kingdom^17^	0.029	NA	0.109	0.284
Netherlands & Germans^21^	NA	NA	0.07 (HTG) 0.099 (Control)	NA
Indians^22^	0.065	0.146	NA	NA
Kuwait (Current Study)	0.065	0.379	0.087	0.337

MAF: Minor Allele Frequency; HTG: high triglycerides; LTG: low triglycerides.

*Ensembl genome browser 92.

## Conclusion

The findings in the present study confirm the role of genetic variants in the noncoding regions with variation in plasma lipid levels. These variants may play a direct role in exerting their effect either on the expression of the gene directly or through the interaction with other variants. In addition, the identification of a significant association of two SNPs with variation in plasma lipid levels may be specific to the Arab ethnic group represented by the Kuwaiti population. Previous studies have emphasized the role of local ancestry in generating significant association of genetic variants with plasma lipid levels that may be ethnic specific^3,6^. This in turn highlights the importance of identifying ethnic specific genetic variants that can affect lipid metabolism. Once identified with a confirmed effect, either “risk” or “protective”, such variants could be used to form molecular diagnostic panels for screening for dyslipidemia and associated clinical diseases. The findings in this study suggest that rs1121923 and rs258 should be considered for such panels and for the estimation of “risk” to dyslipidemia in admixed populations. Although no significant findings were observed for rs328 and rs13702, despite numerous reports on their association, the results from this study on those two variants are inconclusive. This is probably due to the fact that the cohort in the present study did not include patients with confirmed clinical diagnosis of dyslipidemia, T2DM and/or CHD. It is important to point out that one of the strengths of the study was to use “common” variants to assess the effect of *LPL* on plasma lipid levels in a reasonably sized cohort whilst allowing the identification of homozygosity for the minor alleles and the analysis of its frequency distribution. The other strength was the selection of four variants across the *LPL* gene locus that were not in linkage disequilibrium to demonstrate the effect of the various regions may play in altering LPL activity and subsequent regulation of plasma lipid levels. It is strongly recommended that the functional role of rs258 in protein binding of transcription factors be investigated.

Our study highlights the complex interaction between coding and non-coding regions, and the summative or subtractive effects different variants may have on an outcome such as plasma lipid levels. This may explain the ambiguous and sometimes conflicting results obtained by different studies when dealing with different populations.

## Methods

### Sample Description and Biochemical Parameters

This study was approved (Reference number: VDR/JC/256) by the Joint Committee for the Protection of Human Subjects in Research (Health Sciences Center, Kuwait University and Kuwait Institute for Medical Specializations) and conducted in accordance with the procedures set in the Helsinki guidelines. Each study subject was required to give voluntary informed consent to participate in the study and provide a blood sample. A total of 702 blood samples were collected in EDTA tubes by a certified nurse in the biochemistry laboratory at several medical clinics/hospitals in Kuwait during the period from 2012 to 2016. The subjects were randomly recruited with the following inclusion criteria: age above 18 years, willingness to provide blood for fasting lipid profile and Kuwaiti nationality (Table 10). The exclusion criteria were confirmed clinical diagnosis of T2DM, CHD, taking any medication that may alter plasma lipid levels and refusal to give informed consent. The diagnosis for T2DM was based on the HbA1c criteria of the American Diabetes Association (American Diabetes Association. Diagnosis and classification of diabetes mellitus^34^. Using the criteria, HbA1c ≥6.5% (48 mmol/L) was indicative of T2DM. Presence or absence of CHD was determined as previously described^35^. Briefly, subjects with history of myocardial infarction or angina were evaluated with the Rose questionnaire^36^. Subjects without history of CHD were evaluated with resting electrocardiographic (ECG) coded using the Minnesota codes^37^ and CHD was defined as probable ischemia (code 1.1–1.2) or possible ischemia (code 1.3, 4.1–4.4, 5.1–5.3, or 7.1).

**Table 10:** Demographic description of the Kuwaiti cohort (n = 702).

Variable	Subjects
Gender (n)	
Males	282 (40.2%)
Females	420 (59.2%)
Age (yrs)	
Mean ± SE	32.8 ± 0.53
Median [Range]	28 [18–76]
BMI	
Mean ± SE	27.1 ± 0.3
Median (Interquartile Range)	25.7 [16.4–62.4]
TC (mmol/L)	
Mean ± SE	4.7 ± 0.03
Median	4.6 [2–7.9]
HDL-C (mmol/L)	
Mean ± SE	1.1 ± 0.01
Median	1.1 [0.3–2.75]
LDL-C (mmol/L)	
Mean ± SE	3.1 ± 0.03
Median	3 [0.7–6.3]
VLDL (mmol/L)	
Mean ± SE	0.44 ± 0.01
Median	0.34 [0.06–3.33]
TG (mmol/L)	
Mean ± SE	1.07 ± 0.03
Median	0.84 [0.16–7.33]

### Biochemical Parameters and Lipid Level Determination

Standard plasma lipid parameters (plasma total cholesterol (TC); Triglycerides (TG), HDL-C, low-density lipoproteins (LDL-C) and very low-density lipoprotein (VLDL) were determined on an automated chemistry analyzer (Beckman Unicel DxC 800, Beckman Corporation, Brea, CA, USA) using commercially available reagents. TC was measured using a multi-step enzymatic end point method that breaks down cholesterol into Quinonimine and water. TG was measured using a timed-endpoint method in a sequence of multi-enzyme reactions using glycerol kinase, glycerophosphate oxidase and horseradish peroxidase. In the final step of the reactions, formation of a red quinonimine dye measurement at 520 nm. HDL-C in the sample was released by a detergent which solubilizes only the HDL-C lipoprotein particles. Released HDL-C was reacted with cholesterol esterase and cholesterol oxidase to produce a color product that measured at 560 nm. LDL-C and (VLDL-C were calculated using the Friedewald formula: LDL cholesterol = Total cholesterol – HDL cholesterol – (Total triglyceride ÷ 2.2). Total triglyceride ÷ 2.2 provides a good estimate of VLDL^38^. The reference values used in the present study are those set by the Kuwait Ministry of Health where: TC = 3.0–5.17 mmol/L, TG = 0.40–1.7 mmol/L, HDL-C = 0.91–2.5 mmol/L and LDL-C = 1.8–3.2 mmol/L.

### DNA Extraction and Genotyping

Total genomic DNA was isolated from 5 ml of whole blood using a salt extraction method^39^. All DNA samples were analyzed qualitatively and quantitively for suitability of use in the genotyping assay by Realtime PCR^27^. All suitable DNA samples were standardized to give a final concentration of 10 ng/ul. The genotyping assay for the four selected *LPL* variants (rs1121923; rs258; rs328 and rs13702) was achieved by the Taqman Allele Discrimination Assay with Realtime PCR (ABI 7900HT FAST REAL TIME PCR) on a 10 ng/µl DNA with commercially available primer and probe Kits (Table 11) as described by the manufacturer’s protocol (Thermo fisher Scientific, Applied Biosystems).

**Table 11:** List of the *LPL* variants and their primer-probe kits (Thermo fisher Scientific, Applied Biosystems) selected for the genetic association with plasma lipid levels in a Kuwaiti Cohort.

Sl. No.	SNP ID #	Marker name	Context Sequence [VIC/FAM]:
1	rs1121923	LPL-C___8804861_10	ACACCAAACTGGTGGGACAGGATGT**[A/G]** GCCCGGTTTATCAACTGGATGGAGG
2	rs258	C___1842994_10	TACTGGAACAGAAGATGTTAATTAG**[C/G]** ATAAATCTTCCAAAATGTTCAGAAC
3	rs328	LPL-C____901792_1_	CATGACAAGTCTCTGAATAAGAAGT**[C/G]** AGGCTGGTGAGCATTCTGGGCTAAA
4	rs13702	C___9639448_10	GGAACTCTGGCTCCGAAAAACTTTG**[C/T]** TATATATATCAAGGATGTTCTGGCT

### Genotyping, Hardy-Weinberg Equilibrium and Linkage Disequilibrium

The genotypes for each sample (Supplementary Table S1) was relatively simple to determine based on the SDS plots generated following the completion of Realtime PCR. Each sample was genotyped as homozygote for the wildtype allele (WW), heterozygous (WM) or homozygous for the minor allele (MM). Based on the generated genotypes, genotype and allele frequencies were estimated using a simple gene counting method in which the minor allele frequency (MAF) was determined for each variant in the cohort. Deviation from Hardy-Weinberg Equilibrium was estimated using the web-based calculator available at http://www.tufts.edu/.

Haploview (version 4.2.) based on the method described by Gabriel *et al*. (2002) was used to determine linkage disequilibrium (LD) patterns and haplotype blocks. LD was measured as r-squared value (r^2^) by an estimation of each pair-wise combination of SNPs. R^2^ values greater than 0.8 indicate a significant LD between two loci whereas a value of 0 indicates that the two loci are in linkage equilibrium. All pairs of markers (SNPs) following one of those conditions were said to be informative markers, whereas other markers falling outside that value were said to be non-informative. A haplotype block was then created if 95% of informative comparisons were in strong LD.

### Genetic Association and Regression Analysis

Kruskal-Wallis ANOVA was used to analyze differences in mean between genotypes and lipid levels. The values were reported as mean ± standard error. This was followed by linear regression to assess the association of the four variants with lipid levels after controlling for age, gender and BMI. The values of the regression analysis were represented as beta (B) coefficient and 95% confidence intervals (CI). Multivariate analysis using linear regression to assess predictor factors was followed for significant lipid variables. Normality was assessed using Kolmogorov-Smirnov test. All lipid parameters were log-transformed for their association with *LPL* variants to ensure an approximate normal distribution. After Bonferroni correction for multiple testing of the four SNPs, the modified significance level = 0.5/4 = 0.0125. All statistical analyses reported in this study were performed using both the Statistical Package for the Social Sciences software (version 23; SPSS Inc., Chicago, IL, USA) and “SNPassoc” package from R software (R Stats Package, Version 3.3.0) where appropriate.

### Ethical Approval

This study was approved (Reference number: VDR/JC/256) by the Joint Committee for the Protection of Human Subjects in Research (Health Sciences Center, Kuwait University and Kuwait Institute for Medical Specializations) in accordance to the procedures set and that are based on the Helsinki guidelines. The sample and medical data collection protocol and informed consents used were in accordance with the revised version (2000) of the 1975 Helsinki guidelines. Informed consent in this study was obtained from each participant.

## Supplementary information

Genetic association of LPL rs1121923 and rs258 with plasma TG and VLDL levels Al-Bustan S; Al-Serri A, Alnaqeeb M; Annice B; Mojiminiyi O - Supplementary Table S1: LPL variants genotypes in the Kuwai

## Data Availability

The raw genotypic data is provided in Supplementary Table S1. Any additional may be available upon request.
