# Supplementary material for: Genetic association of LPL rs1121923 and rs258 with plasma TG and VLDL levels
Source: Sci Rep. 2019 Apr 3;9:5572. doi: 10.1038/s41598-019-42021-3 (PMC6447523; doi:10.1038/s41598-019-42021-3)
Supplement: Supplementary file 1 — Genetic association of LPL rs1121923 and rs258 with plasma TG and VLDL levels Al-Bustan S; Al-Serri A, Alnaqeeb M; Annice B; Mojiminiyi O - Supplementary Table S1: LPL variants genotypes in the Kuwai [file 41598_2019_42021_MOESM1_ESM.pdf]

Supplementary Information

Supplementary Table S1: LPL variants genotypes in the Kuwaiti cohort (n=702)

Genetic association of LPL rs1121923 and rs258 with plasma TG and VLDL levels

Al-Bustan S; Al-Serri A, Alnaqeeb M; Annice B; Mojiminiyi O

1Department of Biological Sciences, Faculty of Science, Kuwait University; 2Unit of Human Genet
Faculty of Medicine, Kuwait University, 3Mubark Al-Kabeer Hospital, Ministry of Health, Kuwait

Sl. No.	Sample No.	rs1121923	rs328	rs13702	rs258
1	KWN 004	G/G	C/C	T/T	G/G
2	KWN 005	G/G	C/C	C/C	G/G
3	KWN 008	G/G	C/G	T/C	G/C
4	KWN 009	G/G	C/C	T/T	G/C
5	KWN 012	G/G	C/C	T/T	G/G
6	KWN 014	G/G	C/C	T/C	G/C
7	KWN 015	G/A	C/G	T/C	G/C
8	KWN 016	G/A	C/C	C/C	C/C
9	KWN 017	G/G	C/C	T/T	G/G
10	KWN 018	G/G	C/C	T/T	G/C
11	KWN 024	G/A	C/C	T/C	G/C
12	KWN 025	G/G	C/C	T/T	G/C
13	KWN 027	G/G	C/C	T/T	G/C
14	KWN 030	G/G	C/C	T/C	G/C
15	KWN 031	G/G	C/G	T/C	G/C
16	KWN 035	G/G	C/C	T/T	G/C
17	KWN 037	G/A	C/C	C/C	G/C
18	KWN 039	G/G	C/C	T/T	C/C
19	KWN 041	G/G	C/C	T/T	G/C
20	KWN 042	G/G	C/C	T/T	G/G
21	KWN 043	G/G	C/C	T/T	G/C
22	KWN 044	G/G	C/C	T/T	G/C
23	KWN 045	G/A	C/C	C/C	C/C
24	KWN 046	G/G	C/C	T/C	G/G
25	KWN 047	G/G	C/G	T/C	C/C
26	KWN 048	G/G	C/C	T/C	G/C
27	KWN 049	G/A	C/C	T/T	G/C

28	KWN 051	G/G	C/C	T/T	G/G
29	KWN 053	G/A	C/C	T/C	G/C
30	KWN 054	G/G	C/C	T/T	G/C
31	KWN 055	G/G	C/C	T/T	G/G
32	KWN 056	G/G	C/C	T/T	G/G
33	KWN 057	G/G	C/C	T/C	G/G
34	KWN 059	G/A	C/G	C/C	C/C
35	KWN 063	G/G	C/C	T/T	G/G
36	KWN 064	G/G	C/C	T/C	G/C
37	KWN 065	G/G	C/C	T/C	G/C
38	KWN 066	G/G	C/C	T/T	G/C
39	KWN 067	G/G	C/C	T/T	G/G
40	KWN 068	G/G	C/C	T/C	G/C
41	KWN 070	G/G	C/C	T/T	G/G
42	KWN 071	G/G	C/C	T/T	G/G
43	KWN 072	G/G	C/C	T/T	G/G
44	KWN 073	G/A	C/C	T/C	G/C
45	KWN 074	G/G	C/G	T/C	G/C
46	KWN 075	G/G	C/G	C/C	G/C
47	KWN 076	G/G	C/C	T/C	G/C
48	KWN 077	G/G	C/G	T/C	C/C
49	KWN 078	G/G	C/C	T/T	G/G
50	KWN 080	G/G	C/C	T/T	G/G
51	KWN 081	G/G	C/C	T/T	G/C
52	KWN 082	G/G	C/C	T/C	C/C
53	KWN 084	G/G	C/C	T/C	G/G
54	KWN 085	G/G	C/C	T/T	G/G
55	KWN 086	G/G	C/C	T/C	G/C
56	KWN 089	G/G	C/C	T/T	C/C

57	KWN 090	G/G	C/C	T/C	G/C
58	KWN 091	G/G	C/C	T/T	G/G
59	KWN 093	G/G	C/C	T/C	G/G
60	KWN 094	G/G	C/C	T/T	G/G
61	KWN 097	G/G	C/C	C/C	G/C
62	KWN 100	G/G	C/C	T/T	G/G
63	KWN 101	G/G	C/C	T/T	G/G
64	KWN 125	G/G	C/C	T/T	G/G
65	KWN 127	G/G	C/G	C/C	C/C
66	KWN 128	G/G	C/C	T/T	G/C
67	KWN 132	G/A	C/G	T/C	G/C
68	KWN 133	G/A	C/C	T/C	C/C
69	KWN 134	G/G	C/C	C/C	C/C
70	KWN 135	G/G	C/C	C/C	G/C
71	KWN 136	G/G	C/C	C/C	C/C
72	KWN 137	G/G	C/C	T/T	G/C
73	KWN 139	G/G	C/C	T/T	G/G
74	KWN 140	G/G	C/C	T/C	G/C
75	KWN 141	G/G	C/C	T/T	G/G
76	KWN 143	G/G	C/C	T/C	G/C
77	KWN 144	G/G	C/C	T/T	G/G
78	KWN 146	G/A	C/C	T/C	C/C
79	KWN 147	G/G	C/C	T/C	G/C
80	KWN 148	G/G	C/C	T/T	G/G
81	KWN 149	G/G	C/C	T/T	G/G
82	KWN 151	G/G	C/C	T/T	C/C
83	KWN 152	G/G	C/C	T/T	G/G
84	KWN 153	G/G	C/C	T/C	G/G
85	KWN 154	G/A	C/C	C/C	G/C

86	KWN 155	G/G	C/G	T/C	G/C
87	KWN 156	G/G	C/C	T/C	G/G
88	KWN 157	G/G	C/C	T/T	G/G
89	KWN 158	G/G	C/C	T/C	G/C
90	KWN 159	G/G	C/C	T/C	G/C
91	KWN 160	G/G	C/C	T/T	G/G
92	KWN 161	G/G	C/C	T/T	G/G
93	KWN 162	G/G	C/C	T/T	G/G
94	KWN 163	G/G	C/C	T/C	G/C
95	KWN 164	G/G	C/C	T/T	G/C
96	KWN 165	G/G	C/C	C/C	G/C
97	KWN 166	G/G	C/C	T/T	G/G
98	KWN 167	G/A	C/G	T/C	C/C
99	KWN 168	G/G	C/G	C/C	G/G
100	KWN 169	G/A	C/C	T/C	G/C
101	KWN170	G/G	C/G	T/C	G/C
102	KWN 171	G/G	C/C	T/C	G/G
103	KWN 172	G/G	C/C	C/C	G/G
104	KWN 173	G/G	C/G	C/C	G/C
105	KWN 174	G/G	C/C	T/C	G/G
106	KWN 175	G/G	C/G	C/C	C/C
107	KWN 177	G/G	C/C	T/T	G/G
108	KWN 178	G/G	C/G	T/C	G/C
109	KWN 198	G/G	C/C	T/T	G/C
110	KWN 199	G/G	C/C	T/T	G/C
111	KWN 202	G/G	C/C	T/T	C/C
112	KWN 204	G/G	C/C	T/T	G/C
113	KWN 205	G/G	C/G	C/C	C/C
114	KWN 206	G/G	C/C	T/T	G/G

115	KWN 207	G/G	C/C	T/C	G/G
116	KWN 208	G/G	C/C	T/C	G/G
117	KWN 209	G/G	C/C	T/T	G/G
118	KWN 210	G/G	C/C	T/C	G/G
119	KWN 211	G/G	C/C	T/T	C/C
120	KWN 212	G/G	C/C	T/C	G/C
121	KWN 214	G/G	C/C	T/C	G/G
122	KWN 215	G/A	C/C	T/C	G/C
123	KWN 216	G/G	C/C	T/T	G/G
124	KWN 217	G/A	C/C	T/T	C/C
125	KWN 218	G/G	C/C	T/T	G/G
126	KWN 219	G/G	C/C	T/T	C/C
127	KWN 220	G/G	C/C	C/C	G/G
128	KWN 221	G/A	C/C	C/C	G/C
129	KWN 223	G/G	C/C	T/T	G/G
130	KWN 225	G/G	C/C	T/C	G/C
131	KWN 227	G/G	C/C	T/T	G/G
132	KWN 229	G/A	C/C	T/C	G/C
133	KWN 230	G/G	C/C	T/C	G/C
134	KWN 231	G/G	C/C	T/T	G/G
135	KWN 232	G/G	C/C	T/T	G/G
136	KWN 233	G/G	C/C	T/C	G/C
137	KWN 234	G/G	C/C	T/T	G/C
138	KWN 235	G/G	C/C	T/T	G/C
139	KWN 236	G/G	C/C	T/C	G/G
140	KWN 237	G/G	C/C	T/C	G/G
141	KWN 238	G/G	C/C	T/C	G/C
142	KWN 239	G/G	C/C	T/C	G/G
143	KWN 240	G/A	C/C	T/C	G/C

144	KWN 241	G/G	C/C	T/C	C/C
145	KWN 243	G/G	C/C	T/T	G/C
146	KWN 244	G/G	C/C	T/T	G/G
147	KWN 245	G/G	C/C	T/T	G/G
148	KWN 246	G/A	C/G	T/C	C/C
149	KWN 247	G/G	C/G	C/C	C/C
150	KWN248	G/G	C/C	T/C	C/C
151	KWN249	G/G	C/C	T/T	G/C
152	KWN250	G/G	C/C	T/T	G/C
153	KWN251	G/G	C/G	T/C	G/C
154	KWN252	G/G	C/C	T/T	G/G
155	KWN253	G/A	C/C	C/C	G/C
156	KWN254	G/G	C/G	T/C	G/C
157	KWN255	G/A	C/G	T/C	G/C
158	KWN256	G/G	C/C	T/C	G/G
159	KWN258	G/A	C/C	T/C	G/C
160	KWN259	G/G	C/G	T/C	G/C
161	KWN260	G/G	C/G	T/C	G/C
162	KWN261	G/A	C/G	C/C	G/C
163	KWN262	G/G	C/C	C/C	C/C
164	KWN263	G/G	C/C	T/T	G/C
165	KWN264	G/G	C/C	T/T	G/C
166	KWN265	G/G	C/C	T/T	G/C
167	KWN266	G/G	C/C	T/C	G/C
168	KWN267	G/G	C/C	T/T	G/G
169	KWN268	G/G	C/C	T/T	G/G
170	KWN269	G/G	C/C	T/T	G/G
171	KWN270	G/G	C/C	T/C	G/C
172	KWN271	G/G	C/C	T/C	G/G

173	KWN272	G/G	C/C	T/C	G/C
174	KWN273	G/G	C/C	T/T	G/G
175	KWN274	G/G	C/G	T/C	G/C
176	KWN275	G/G	C/C	T/T	G/C
177	KWN276	G/G	C/C	C/C	G/C
178	KWN277	G/G	C/C	T/T	G/C
179	KWN278	G/G	C/C	T/C	G/G
180	KWN279	G/G	C/C	T/T	G/G
181	KWN280	G/G	C/C	T/T	C/C
182	KWN281	G/A	C/C	T/C	G/C
183	KWN282	G/G	C/C	T/T	G/C
184	KWN283	G/A	C/G	T/C	G/C
185	KWN284	G/G	C/C	T/T	G/G
186	KWN285	G/G	C/G	T/C	G/C
187	KWN286	G/G	C/C	T/C	G/G
188	KWN287	G/A	C/C	T/C	C/C
189	KWN288	G/G	C/C	C/C	G/G
190	KWN289	G/G	C/C	T/C	G/C
191	KWN290	G/G	C/C	T/C	G/C
192	KWN291	G/A	C/C	T/C	G/C
193	KWN292	G/G	C/C	T/T	G/G
194	KWN293	G/G	C/C	T/C	G/G
195	KWN294	G/G	C/C	T/C	G/C
196	KWN295	G/G	C/C	T/C	C/C
197	KWN296	G/G	C/C	T/C	G/C
198	KWN297	G/G	C/C	T/C	G/C
199	KWN298	G/G	C/C	T/T	G/G
200	KWN299	G/G	C/C	C/C	G/G
201	KWN300	G/G	C/C	T/T	G/G

202	KWN301	G/G	C/C	T/T	G/G
203	KWN302	G/G	C/C	T/C	G/C
204	KWN303	G/G	C/C	T/C	G/G
205	KWN304	G/G	C/C	T/T	G/C
206	KWN305	G/G	C/C	T/T	G/G
207	KWN306	G/G	C/C	T/T	G/C
208	KWN307	G/G	C/C	T/C	G/C
209	KWN308	G/G	C/C	T/T	G/G
210	KWN309	G/A	C/G	T/C	G/C
211	KWN310	G/G	C/G	C/C	G/C
212	KWN311	G/G	C/G	C/C	G/C
213	KWN312	G/G	C/C	T/T	G/G
214	KWN313	G/G	C/C	T/T	G/C
215	KWN314	G/G	C/G	C/C	G/C
216	KWN315	G/G	C/C	T/C	C/C
217	KWN316	G/G	C/C	T/T	G/G
218	KWN317	G/G	C/C	T/T	G/C
219	KWN318	G/G	C/C	T/T	C/C
220	KWN319	G/G	C/C	T/C	G/G
221	KWN320	G/G	C/C	T/C	G/C
222	KWN321	G/G	C/C	T/C	G/C
223	KWN322	G/G	C/C	T/C	G/G
224	KWN323	G/G	C/C	T/C	G/C
225	KWN324	G/A	C/C	T/C	C/C
226	KWN325	G/G	C/C	T/T	G/C
227	KWN326	G/G	C/C	T/T	G/G
228	KWN327	G/G	C/C	T/T	G/G
229	KWN328	G/G	C/G	T/C	C/C
230	KWN329	G/A	C/C	T/T	C/C

231	KWN330	G/G	C/C	C/C	G/C
232	KWN331	G/G	C/C	T/T	G/G
233	KWN332	G/G	C/C	T/C	G/C
234	KWN333	G/G	C/C	C/C	C/C
235	KWN334	G/G	C/C	C/C	G/C
236	KWN335	G/G	C/C	T/C	G/C
237	KWN336	G/G	C/G	T/C	G/C
238	KWN337	G/G	C/C	T/T	G/G
239	KWN338	G/G	C/C	T/T	G/C
240	KWN339	G/G	C/C	T/C	G/C
241	KWN340	G/G	C/C	T/C	G/C
242	KWN341	G/G	C/C	T/C	G/C
243	KWN342	G/G	C/G	T/C	G/C
244	KWN343	G/G	C/C	T/C	G/G
245	KWN344	G/G	C/C	T/C	G/G
246	KWN345	G/G	C/C	T/T	G/G
247	KWN346	G/G	C/C	T/T	G/G
248	KWN347	G/G	C/C	T/C	G/C
249	KWN348	A/A	C/G	T/C	G/C
250	KWN349	G/G	C/C	T/T	G/G
251	KWN350	G/A	C/C	T/C	C/C
252	KWN351	G/G	C/C	T/C	G/C
253	KWN352	G/G	C/C	T/T	G/C
254	KWN353	G/G	C/G	C/C	G/C
255	KWN354	G/A	C/C	T/T	G/C
256	KWN355	G/G	C/C	T/T	G/C
257	KWN356	G/A	C/G	T/C	C/C
258	KWN357	G/G	C/G	T/C	G/C
259	KWN358	G/G	C/C	T/C	G/C

260	KWN359	G/G	C/C	C/C	G/G
261	KWN360	G/G	C/C	T/T	G/G
262	KWN361	G/G	C/C	T/T	G/G
263	KWN362	G/G	C/C	T/T	G/C
264	KWN363	G/G	C/C	T/C	G/C
265	KWN364	G/G	C/C	T/T	G/C
266	KWN365	G/A	C/G	T/T	C/C
267	KWN366	G/G	C/C	T/T	G/G
268	KWN367	G/G	C/C	T/C	G/C
269	KWN368	G/G	C/C	T/T	G/G
270	KWN369	G/G	C/C	T/T	G/G
271	KWN370	G/G	C/G	T/C	G/C
272	KWN371	G/G	C/C	T/C	C/C
273	KWN372	G/G	C/C	C/C	G/G
274	KWN373	G/G	C/C	C/C	G/C
275	KWN374	G/G	C/C	T/T	G/G
276	KWN375	G/G	C/C	T/T	G/G
277	KWN376	G/G	C/C	T/C	G/G
278	KWN377	G/G	C/C	T/C	G/G
279	KWN378	G/G	C/G	C/C	G/C
280	KWN379	G/G	C/C	T/T	G/G
281	KWN380	G/A	C/C	T/T	G/C
282	KWN381	G/G	C/C	C/C	G/G
283	KWN382	G/G	C/C	T/T	G/G
284	KWN383	G/G	C/C	C/C	G/C
285	KWN384	G/G	C/C	T/C	G/G
286	KWN385	G/G	C/C	T/T	G/G
287	KWN386	G/G	C/G	T/C	G/C
288	KWN387	G/G	C/G	T/C	G/C

289	KWN388	G/G	C/C	T/C	G/G
290	KWN389	G/G	C/G	T/C	C/C
291	KWN390	G/G	C/C	T/T	G/G
292	KWN391	G/G	C/G	C/C	G/C
293	KWN392	G/G	C/C	T/C	G/G
294	KWN393	G/G	C/C	T/T	G/G
295	KWN394	G/G	C/C	T/T	G/G
296	KWN395	G/G	C/G	T/C	G/C
297	KWN396	G/G	C/C	C/C	G/C
298	KWN397	G/G	C/C	T/T	C/C
299	KWN398	G/G	C/C	T/C	G/C
300	KWN399	G/G	C/C	T/T	G/G
301	KWN400	G/G	C/C	T/T	G/C
302	KWN401	G/G	C/C	T/T	C/C
303	KWN402	G/G	C/C	T/C	C/C
304	KWN403	G/G	C/C	T/T	G/G
305	KWN404	G/G	C/C	T/T	G/G
306	KWN405	G/G	C/C	T/T	G/C
307	KWN406	G/G	C/C	T/T	G/G
308	KWN407	G/G	C/C	T/C	C/C
309	KWN408	G/G	C/C	T/T	G/C
310	KWN409	G/G	C/C	T/T	G/G
311	KWN410	G/G	C/C	T/T	G/G
312	KWN411	G/G	C/C	T/T	G/C
313	KWN412	G/G	C/G	T/C	G/G
314	KWN413	G/G	C/C	T/T	G/G
315	KWN414	G/G	C/C	T/C	G/G
316	KWN415	G/A	C/G	T/C	G/C
317	KWN416	G/G	C/G	T/C	G/C

318	KWN417	G/G	C/C	T/C	G/G
319	KWN418	G/G	C/G	T/C	G/C
320	KWN419	G/G	C/C	T/T	C/C
321	KWN420	G/G	C/C	T/T	G/G
322	KWN421	G/G	C/C	T/C	G/G
323	KWN422	G/G	C/C	T/C	G/C
324	KWN423	G/G	C/G	T/C	C/C
325	KWN424	G/A	C/G	T/C	G/C
326	KWN425	G/G	C/C	T/C	G/G
327	KWN426	G/G	C/C	C/C	G/G
328	KWN427	G/G	C/C	T/T	G/G
329	KWN428	G/A	C/C	T/C	G/C
330	KWN429	G/G	C/C	T/C	G/C
331	KWN430	G/A	C/C	T/C	G/C
332	KWN431	G/G	C/C	T/T	G/C
333	KWN432	G/G	C/G	T/C	G/C
334	KWN433	G/A	G/G	C/C	C/C
335	KWN434	G/G	C/C	T/C	G/C
336	KWN435	G/G	C/C	C/C	C/C
337	KWN436	G/G	C/C	T/T	G/G
338	KWN437	G/G	C/C	T/T	G/C
339	KWN438	G/G	C/C	T/T	G/C
340	KWN439	G/G	C/C	T/T	G/C
341	KWN440	G/G	C/C	T/T	G/G
342	KWN441	G/G	C/C	C/C	G/G
343	KWN442	G/G	C/C	T/T	G/G
344	KWN443	A/A	C/C	C/C	C/C
345	KWN444	G/G	C/C	T/T	G/C
346	KWN445	G/G	C/C	T/T	G/G

347	KWN446	G/G	C/C	T/T	G/C
348	KWN447	G/G	C/C	T/T	G/G
349	KWN448	G/G	C/C	T/T	G/G
350	KWN449	G/G	C/C	T/C	G/C
351	KWN450	G/G	C/G	T/C	G/C
352	KWN451	G/G	C/C	T/T	G/C
353	KWN452	G/G	C/C	T/C	C/C
354	KWN454	G/A	C/C	T/C	G/C
355	KWN455	G/A	C/C	C/C	C/C
356	KWN456	G/G	C/C	T/T	G/C
357	KWN457	G/G	C/C	T/T	G/G
358	KWN458	G/G	C/C	T/C	G/G
359	KWN459	G/G	C/C	T/T	G/C
360	KWN460	G/G	C/C	T/T	G/G
361	KWN461	G/G	C/C	T/T	G/G
362	KWN462	G/A	C/C	T/C	G/C
363	KWN463	G/G	C/C	T/C	G/G
364	KWN464	G/G	C/C	T/T	G/G
365	KWN465	G/A	C/G	T/C	G/C
366	KWN466	G/G	C/C	T/T	G/C
367	KWN467	G/G	C/C	T/T	G/C
368	KWN468	G/G	C/C	T/C	G/G
369	KWN469	G/G	C/C	T/T	G/G
370	KWN470	G/G	C/G	T/C	G/C
371	KWN472	G/G	C/C	T/C	C/C
372	KWN473	G/G	C/C	T/T	G/G
373	KWN474	G/G	C/C	T/T	G/G
374	KWN475	G/G	C/C	T/T	G/G
375	KWN476	G/G	C/G	T/C	C/C

376	KWN477	G/G	C/C	T/T	G/G
377	KWN478	G/G	C/C	T/C	G/G
378	KWN479	G/G	C/C	T/T	G/C
379	KWN480	G/G	C/C	T/T	G/G
380	KWN481	G/G	C/C	T/T	G/G
381	KWN482	G/G	C/C	T/T	G/G
382	KWN483	G/G	C/C	T/T	G/C
383	KWN484	G/G	C/C	T/T	G/C
384	KWN485	G/G	C/C	T/C	G/C
385	KWN486	G/G	C/C	T/T	G/C
386	KWN487	G/G	C/C	T/C	G/C
387	KWN489	G/G	C/C	T/T	G/C
388	KWN490	G/G	C/G	C/C	C/C
389	KWN491	G/G	C/C	T/C	G/G
390	KWN492	G/G	C/G	T/C	G/C
391	KWN493	G/G	C/C	T/T	G/G
392	KWN494	G/G	C/C	T/C	C/C
393	KWN495	G/G	C/C	T/T	G/G
394	KWN496	G/G	C/C	T/C	G/G
395	KWN497	G/A	C/C	T/C	G/C
396	KWN501	G/G	C/C	T/C	G/G
397	KWN502	G/G	C/C	T/C	G/G
398	KWN503	G/G	C/C	T/T	G/C
399	KWN505	G/G	C/C	T/T	G/C
400	KWN506	G/G	C/C	T/C	G/C
401	KWN508	G/G	C/C	T/C	G/G
402	KWN509	G/G	C/C	T/T	G/G
403	KWN512	G/G	C/C	T/C	G/C
404	KWN513	G/G	C/C	T/C	G/G

405	KWN514	G/G	C/G	C/C	C/C
406	KWN516	G/G	C/C	T/C	G/C
407	KWN517	G/G	C/C	T/T	G/G
408	KWN523	G/G	C/C	T/C	G/C
409	KWN525	G/G	C/C	T/T	G/C
410	KWN526	G/G	C/C	T/T	G/C
411	KWN528	G/A	C/C	T/C	C/C
412	KWN529	G/A	C/C	T/C	G/C
413	KWN530	G/G	C/C	T/C	G/G
414	KWN532	G/G	C/C	T/T	G/G
415	KWN533	G/G	G/G	C/C	C/C
416	KWN534	G/G	C/G	T/C	C/C
417	KWN535	G/G	C/C	T/C	G/G
418	KWN537	G/G	C/G	T/C	C/C
419	KWN542	G/G	C/C	T/T	G/C
420	KWN547	G/A	C/C	T/C	G/C
421	KWN549	G/G	C/C	T/C	G/C
422	KWN553	G/G	C/C	T/T	G/G
423	KWN554	G/G	C/C	T/C	G/G
424	KWN566	G/A	C/G	T/C	G/C
425	KWN568	G/G	C/C	T/C	G/G
426	KWN 572	G/G	C/C	T/C	G/G
427	KWN 574	G/G	C/C	T/C	G/G
428	KWN 585	G/G	C/C	T/T	G/G
429	KWN 588	G/G	C/C	T/T	G/G
430	KWN 590	G/G	C/C	T/T	G/G
431	KWN 591	G/G	C/C	T/C	G/G
432	KWN 592	G/G	C/C	T/T	G/C
433	KWN 593	G/G	C/C	T/T	G/C

434	KWN 594	G/G	C/C	T/T	G/G
435	KWN 595	G/A	C/C	T/T	G/C
436	KWN 596	G/G	C/C	T/C	G/G
437	KWN 598	G/A	C/G	T/C	G/C
438	KWN 599	G/G	C/C	T/T	G/C
439	KWN 602	G/G	C/C	T/T	G/C
440	KWN 603	G/G	C/C	T/C	G/G
441	KWN 604	G/G	C/C	T/C	G/C
442	KWN 605	G/G	C/C	T/C	G/C
443	KWN 606	G/G	C/C	T/C	G/G
444	KWN 608	G/A	C/C	T/C	G/C
445	KWN 610	G/G	C/C	T/T	G/G
446	KWN 612	G/G	C/C	T/T	G/C
447	KWN 613	G/G	C/C	T/T	G/G
448	KWN 616	G/G	C/C	T/T	G/G
449	KWN 618	G/G	C/C	T/C	G/G
450	KWN 619	G/G	C/C	T/T	G/G
451	KWN 622	G/A	C/C	T/C	G/C
452	KWN 624	G/A	C/G	C/C	C/C
453	KWN 625	G/G	C/C	T/T	G/G
454	KWN 626	G/G	C/C	T/C	G/C
455	KWN 627	G/G	C/G	T/C	G/C
456	KWN 628	G/G	C/C	T/C	G/G
457	KWN 633	G/G	C/C	T/T	G/C
458	KWN 634	G/G	C/G	T/C	G/C
459	KWN 635	G/G	C/C	T/T	G/G
460	KWN 638	G/G	C/C	T/T	G/G
461	KWN 651	G/A	C/G	T/C	G/C
462	KWN 652	G/G	C/C	T/T	G/G

463	KWN 653	G/G	C/C	T/C	G/C
464	KWN 665	G/G	C/G	T/C	G/C
465	KWN 667	G/G	C/G	T/C	G/C
466	KWN 669	G/G	C/C	T/C	G/G
467	KWN 670	G/G	C/C	T/C	G/C
468	KWN 671	G/G	C/G	T/C	C/C
469	KWN 672	G/G	C/C	T/C	G/C
470	KWN 673	G/G	C/C	T/T	G/C
471	KWN 676	G/G	C/C	T/C	G/G
472	KWN 677	G/G	C/C	T/T	G/G
473	KWN 679	G/G	C/C	T/C	G/C
474	KWN 685	G/G	C/C	T/T	G/G
475	KWN 692	G/G	C/C	T/T	G/G
476	KWN 694	G/G	C/C	T/T	G/G
477	KWN 697	G/G	C/C	T/T	G/C
478	KWN 699	G/A	C/C	T/T	C/C
479	KWN 700	G/G	C/C	T/C	G/C
480	KWN 701	G/G	C/C	T/C	G/G
481	KWN 708	G/G	C/C	T/T	C/C
482	KWN 721	G/G	C/G	T/C	C/C
483	KWN 722	G/G	C/C	T/C	G/G
484	KWN 723	G/G	C/C	T/T	G/G
485	KWN 725	G/G	C/C	T/T	G/G
486	KWN 731	G/G	C/G	T/C	G/C
487	KWN 732	G/G	C/C	T/T	G/C
488	KWN 739	G/G	C/G	T/C	G/C
489	KWN 742	G/G	C/C	T/T	G/G
490	KWN 744	G/A	C/G	C/C	G/C
491	KWN 746	G/G	C/C	T/T	G/G

492	KWN 753	G/G	C/C	T/T	G/G
493	KWN 754	G/G	C/C	T/T	G/G
494	KWN 756	G/G	C/C	T/C	G/C
495	KWN 759	G/G	C/G	T/C	G/G
496	KWN 762	G/G	C/C	T/T	C/C
497	KWN 766	G/G	C/C	T/C	G/C
498	KWN 771	G/G	C/C	T/C	G/G
499	KWN 772	G/G	C/C	T/T	G/C
500	KWN 774	G/G	C/G	T/C	G/C
501	KWN 775	G/A	C/C	T/T	G/C
502	KWN 776	G/G	C/C	T/T	G/C
503	KWN 777	G/A	C/C	C/C	C/C
504	KWN 778	G/G	C/C	T/C	G/G
505	KWN 779	G/A	C/C	T/C	G/C
506	KWN 780	G/G	C/G	T/C	G/C
507	KWN 782	G/G	C/C	T/T	G/C
508	KWN 787	G/G	C/C	C/C	G/G
509	KWN 788	G/G	C/C	T/T	G/G
510	KWN 789	G/G	C/C	T/T	C/C
511	KWN 794	G/G	C/G	T/C	G/C
512	KWN 795	G/G	C/C	T/T	G/G
513	KWN 799	G/G	C/C	T/C	C/C
514	KWN 800	G/G	C/G	T/C	G/C
515	KWN 801	G/A	C/G	T/C	G/C
516	KWN 802	G/G	C/C	T/C	G/C
517	KWN 804	G/G	C/C	T/T	G/G
518	KWN 805	G/G	C/C	T/C	C/C
519	KWN 806	G/G	C/C	T/C	G/C
520	KWN 807	G/G	C/C	T/T	G/C

521	KWN 808	G/A	C/C	T/T	G/C
522	KWN 809	G/G	C/C	T/C	G/C
523	KWN 810	G/G	C/C	T/T	G/C
524	KWN 811	G/G	C/C	T/T	G/G
525	KWN 812	G/G	C/G	T/C	C/C
526	KWN 813	G/G	C/C	C/C	G/C
527	KWN 814	G/G	C/C	C/C	G/G
528	KWN 815	G/G	C/G	C/C	G/G
529	KWN 816	G/G	C/C	T/T	G/G
530	KWN 817	G/G	C/C	T/T	G/C
531	KWN 818	G/G	C/C	C/C	C/C
532	KWN 819	G/G	C/C	T/T	G/C
533	KWN 820	G/G	C/G	C/C	G/C
534	KWN 821	G/G	C/C	T/T	C/C
535	KWN 822	G/G	C/C	C/C	G/G
536	KWN 823	G/G	C/C	C/C	C/C
537	KWN 824	G/G	C/C	C/C	G/C
538	KWN 825	G/G	C/C	T/T	G/G
539	KWN 826	G/G	C/G	T/C	C/C
540	KWN 827	G/G	C/C	T/T	G/G
541	KWN 828	G/G	C/C	T/T	G/G
542	KWN 829	G/G	C/C	T/T	G/G
543	KWN 831	G/G	C/C	T/T	G/G
544	KWN 832	G/G	C/C	T/T	C/C
545	KWN 833	G/G	C/C	C/C	G/C
546	KWN 834	G/G	C/C	T/C	G/C
547	KWN 835	G/G	C/C	T/T	G/C
548	KWN 836	G/A	C/C	C/C	G/C
549	KWN 837	G/G	C/C	T/C	G/G

550	KWN 838	G/G	C/C	T/T	G/G
551	KWN 839	G/G	C/G	T/C	G/C
552	KWN 841	G/G	C/C	C/C	G/G
553	KWN 842	G/G	C/C	C/C	C/C
554	KWN 843	G/G	C/G	T/C	G/C
555	KWN 845	G/G	C/C	T/C	G/C
556	KWN 846	G/G	C/C	T/C	G/C
557	KWN 847	G/G	C/G	C/C	G/G
558	KWN 848	G/G	C/C	T/C	G/G
559	KWN 849	G/G	C/C	T/T	G/G
560	KWN 850	G/G	C/C	T/T	G/C
561	KWN 851	G/G	C/C	T/C	G/G
562	KWN 852	G/G	C/G	C/C	C/C
563	KWN 853	G/G	C/G	C/C	C/C
564	KWN 855	G/G	C/C	T/T	G/G
565	KWN 856	G/G	C/C	T/T	G/G
566	KWN 857	G/A	C/G	T/C	C/C
567	KWN 858	G/G	C/C	T/T	G/C
568	KWN 859	G/G	C/G	C/C	G/C
569	KWN 860	G/G	C/G	C/C	G/C
570	KWN 861	G/G	C/C	T/T	G/G
571	KWN 862	G/G	C/C	T/T	G/C
572	KWN 863	G/A	C/C	C/C	G/C
573	KWN 864	G/G	C/C	T/T	G/C
574	KWN 866	G/G	C/C	T/C	G/G
575	KWN 867	G/G	C/C	T/C	C/C
576	KWN 869	G/G	C/C	T/T	G/C
577	KWN 870	G/G	C/C	T/C	G/C
578	KWN 871	G/G	C/C	T/T	G/C

579	KWN 872	G/G	C/C	T/C	G/G
580	KWN 873	G/A	C/C	T/C	C/C
581	KWN 876	G/G	C/C	T/T	C/C
582	KWN 877	G/G	C/C	T/T	C/C
583	KWN 878	G/A	C/C	T/C	C/C
584	KWN 879	G/G	C/C	T/T	G/G
585	KWN 880	G/G	C/C	T/T	G/G
586	KWN 881	G/G	C/C	T/C	G/C
587	KWN 882	G/G	C/C	T/C	G/G
588	KWN 883	G/G	C/C	T/T	G/C
589	KWN 884	G/G	C/C	T/C	G/C
590	KWN 885	G/A	C/C	T/C	G/C
591	KWN 886	G/G	C/C	T/T	G/G
592	KWN 887	G/G	C/C	C/C	G/C
593	KWN 888	G/G	C/C	T/T	G/C
594	KWN 889	G/G	C/C	C/C	G/C
595	KWN 890	G/G	C/C	C/C	G/C
596	KWN 891	G/G	C/C	T/T	G/G
597	KWN 892	G/G	C/C	T/C	G/C
598	KWN 893	G/A	C/C	T/C	G/C
599	KWN 894	G/G	C/C	T/T	G/G
600	KWN 895	G/G	C/C	T/T	G/C
601	KWN 896	G/G	G/G	C/C	C/C
602	KWN 897	G/G	C/C	T/T	G/C
603	KWN 898	G/G	C/C	T/C	G/C
604	KWN 899	G/A	C/G	T/C	G/C
605	KWN 900	A/A	C/G	C/C	C/C
606	KWN 901	G/G	C/C	T/T	G/G
607	KWN 902	G/G	C/C	T/C	G/C

608	KWN 903	G/G	C/C	T/T	G/G
609	KWN 904	G/G	C/C	T/T	G/G
610	KWN 905	G/G	C/C	T/T	G/G
611	KWN 906	G/G	C/C	T/T	G/G
612	KWN 907	G/G	C/C	T/T	G/G
613	KWN 908	G/G	C/G	T/C	G/C
614	KWN 909	G/A	C/C	T/C	C/C
615	KWN 910	G/G	C/C	T/T	G/G
616	KWN 911	G/G	C/C	T/C	G/G
617	KWN 912	G/G	C/G	T/C	G/C
618	KWN 913	G/G	C/C	T/C	C/C
619	KWN 914	G/G	C/C	T/T	G/G
620	KWN 915	G/G	C/C	T/T	G/G
621	KWN 916	G/G	C/C	T/C	G/G
622	KWN 917	G/A	C/G	T/C	G/C
623	KWN 918	G/A	C/C	T/T	G/C
624	KWN 919	G/G	C/C	C/C	G/G
625	KWN 922	G/G	C/C	T/C	G/C
626	KWN 923	G/G	C/C	T/C	G/C
627	KWN 924	G/G	C/C	T/C	G/C
628	KWN 927	G/A	C/G	T/C	G/C
629	KWN 928	G/G	C/C	C/C	C/C
630	KWN 929	G/G	C/C	T/T	G/G
631	KWN 931	G/G	C/C	T/T	G/G
632	KWN 932	G/G	C/C	T/T	G/C
633	KWN 933	G/G	C/C	T/T	G/G
634	KWN 936	G/G	C/C	T/C	G/G
635	KWN 937	G/G	C/C	T/T	C/C
636	KWN 938	G/G	C/G	T/C	C/C

637	KWN 939	G/G	C/G	C/C	C/C
638	KWN 940	G/G	C/C	T/C	G/C
639	KWN 941	G/G	C/C	T/T	G/G
640	KWN 942	G/A	C/C	T/C	G/C
641	KWN 944	G/G	C/C	T/T	G/G
642	KWN 946	G/G	C/G	T/C	G/C
643	KWN 947	G/A	C/C	T/C	C/C
644	KWN 948	G/G	C/C	T/C	G/C
645	KWN 951	G/G	C/G	C/C	G/C
646	KWN 952	G/A	C/C	T/C	C/C
647	KWN 953	G/G	C/C	T/T	G/G
648	KWN 954	G/G	C/C	T/T	G/C
649	KWN 955	G/G	C/C	T/C	G/G
650	KWN 957	G/G	C/C	T/T	G/C
651	KWN 959	G/G	C/C	C/C	G/C
652	KWN 961	G/G	C/G	T/C	G/C
653	KWN 962	G/G	C/C	T/C	G/G
654	KWN 963	G/G	C/G	T/C	G/G
655	KWN 964	G/G	C/C	T/T	G/G
656	KWN 965	G/G	C/C	T/T	G/G
657	KWN 966	G/G	C/G	T/C	C/C
658	KWN 967	G/A	C/C	T/C	C/C
659	KWN 968	G/G	C/C	T/C	G/C
660	KWN 969	G/G	C/C	T/C	G/C
661	KWN 972	G/G	C/C	T/T	G/G
662	KWN 973	G/G	C/C	T/T	C/C
663	KWN 974	G/G	C/G	C/C	C/C
664	KWN 975	G/G	C/C	T/T	G/G
665	KWN 977	G/G	C/C	T/T	G/C

666	KWN 979	G/A	C/C	T/C	C/C
667	KWN 980	G/G	C/C	T/T	C/C
668	KWN 982	G/G	C/G	T/C	G/C
669	KWN 985	G/G	C/C	T/C	G/C
670	KWN 986	G/G	C/C	T/T	G/C
671	KWN 994	G/G	C/C	T/C	G/G
672	KWN 995	G/A	C/G	T/C	C/C
673	KWN 996	G/G	C/C	T/C	G/C
674	KWN 997	G/G	C/C	T/T	G/G
675	KWN 998	G/G	C/C	T/T	G/G
676	KWN 999	G/G	C/C	T/T	G/C
677	KWN 1000	G/G	C/C	T/T	G/G
678	KWN 1001	G/G	C/C	T/T	G/C
679	KWN 1002	G/A	C/G	C/C	C/C
680	KWN 1003	G/G	C/C	T/T	G/G
681	KWN 1004	G/G	C/G	T/C	C/C
682	KWN 1005	G/G	C/C	T/T	G/C
683	KWN 1006	G/G	C/C	T/C	G/G
684	KWN 1008	G/G	C/C	T/T	G/C
685	KWN 1011	G/G	C/C	T/T	G/G
686	KWN 1012	G/G	C/C	T/T	G/G
687	KWN 1013	G/G	C/C	T/C	G/G
688	KWN 1015	G/G	C/C	T/C	G/C
689	KWN 1016	G/G	C/C	T/T	G/G
690	KWN 1017	G/G	C/C	T/T	G/G
691	KWN 1018	G/G	C/C	T/C	G/C
692	KWN 1021	G/G	C/C	T/C	C/C
693	KWN 1022	G/A	C/C	T/C	C/C
694	KWN 1023	G/G	C/G	T/C	C/C

695	KWN 1024	G/G	C/C	T/C	C/C
696	KWN 1026	G/G	C/C	T/C	G/G
697	KWN 1027	G/G	C/G	T/C	C/C
698	KWN 1030	G/G	C/C	T/C	G/C
699	KWN 1033	G/G	C/C	C/C	G/C
700	KWN 1036	G/G	C/G	T/C	G/C
701	KWN 1037	G/A	C/G	T/C	G/C
702	KWN 1038	G/G	C/C	T/T	G/G

tics, Department of Pathology,
